# Different Frequency of Heschl’s Gyrus Duplication Patterns in Neuropsychiatric Disorders: An MRI Study in Bipolar and Major Depressive Disorders

**DOI:** 10.3389/fnhum.2022.917270

**Published:** 2022-06-13

**Authors:** Tsutomu Takahashi, Daiki Sasabayashi, Murat Yücel, Sarah Whittle, Valentina Lorenzetti, Mark Walterfang, Michio Suzuki, Christos Pantelis, Gin S. Malhi, Nicholas B. Allen

**Affiliations:** ^1^Department of Neuropsychiatry, School of Medicine, University of Toyama, Toyama, Japan; ^2^Research Center for Idling Brain Science, University of Toyama, Toyama, Japan; ^3^Brain Park, Turner Institute for Brain and Mental Health, School of Psychological Sciences, Monash University, Clayton, VIC, Australia; ^4^Melbourne Neuropsychiatry Centre, Department of Psychiatry, The University of Melbourne and Melbourne Health, Melbourne, VIC, Australia; ^5^Neuroscience of Addiction and Mental Health Program, Healthy Brain and Mind Research Centre, School of Psychology, Faculty of Health Sciences, Australian Catholic University, Melbourne, VIC, Australia; ^6^Department of Neuropsychiatry, Royal Melbourne Hospital, Melbourne, VIC, Australia; ^7^Florey Institute of Neuroscience and Mental Health, University of Melbourne, Melbourne, VIC, Australia; ^8^North Western Mental Health, Western Hospital Sunshine, St Albans, VIC, Australia; ^9^Academic Department of Psychiatry, Kolling Institute, Northern Clinical School, Faculty of Medicine and Health, The University of Sydney, Sydney, NSW, Australia; ^10^CADE Clinic, Royal North Shore Hospital, Northern Sydney Local Health District, St Leonards, NSW, Australia; ^11^Department of Psychology, University of Oregon, Eugene, OR, United States

**Keywords:** superior temporal gyrus, Heschl’s gyrus, gyrification, major depressive disorder, bipolar disorder

## Abstract

An increased prevalence of duplicated Heschl’s gyrus (HG) has been repeatedly demonstrated in various stages of schizophrenia as a potential neurodevelopmental marker, but it remains unknown whether other neuropsychiatric disorders also exhibit this macroscopic brain feature. The present magnetic resonance imaging study aimed to examine the disease specificity of the established finding of altered HG patterns in schizophrenia by examining independent cohorts of bipolar disorder (BD) and major depressive disorder (MDD). Twenty-six BD patients had a significantly higher prevalence of HG duplication bilaterally compared to 24 age- and sex-matched controls, while their clinical characteristics (e.g., onset age, number of episodes, and medication) did not relate to HG patterns. No significant difference was found for the HG patterns between 56 MDD patients and 33 age- and sex-matched controls, but the patients with a single HG were characterized by more severe depressive/anxiety symptoms compared to those with a duplicated HG. Thus, in keeping with previous findings, the present study suggests that neurodevelopmental pathology associated with gyral formation of the HG during the late gestation period partly overlaps between schizophrenia and BD, but that HG patterns may make a somewhat distinct contribution to the phenomenology of MDD.

## Introduction

Gyrification pattern of Heschl’s gyrus (HG), which includes primary auditory cortex, displays a large inter-individual variability, potentially reflecting cytoarchitectonic development during gestation (Chi et al., 1977; Armstrong et al., 1995) and/or experience-dependent structural plasticity (Zatorre et al., 2012). While functional significance of the HG gyrification patterns has not been fully elucidated, it has been demonstrated that duplicated HG is involved in the neural basis of cognitive skills, such as musicality especially for professional musicians (Schneider et al., 2005; Benner et al., 2017; Turker et al., 2017) and good (Turker et al., 2017) or poor (Leonard et al., 1993, 2001) language learning ability in non-clinical population (reviewed by Marie et al., 2016). HG duplication, which is observed in approximately 30 to 50% of healthy subjects (Leonard et al., 1998; Abdul-Kareem and Sluming, 2008; Marie et al., 2015), is thus thought to be a normal anatomical variant potentially associated with individual differences in cognitive function, but recent magnetic resonance imaging (MRI) studies have suggested that an altered HG gyrification pattern may also be associated with the pathophysiology of neuropsychiatric disorders.

Neuroimaging evidence has demonstrated an association between schizophrenia and macroscopic brain changes (Bakhshi and Chance, 2015; Takahashi and Suzuki, 2018), potentially reflecting early neurodevelopmental pathology (Weinberger, 1987; Insel, 2010). In particular, an increased prevalence of duplicated HG likely exists from the earliest stages of psychosis [e.g., high-risk status (Takahashi et al., 2021c) and at illness onset (Takahashi et al., 2021a)] and is not influenced by medication and illness chronicity (Takahashi et al., 2021b), and may underpin cognitive impairment (Takahashi et al., 2021c) and primary negative symptomatology (Takahashi et al., in submission). These HG findings implicate that altered cytoarchitectonic development of the primary auditory cortex *in utero* may contribute to early neurodevelopmental pathology of schizophrenia. However, the disease specificity of these findings in schizophrenia remains largely unknown. To our knowledge, no studies to date have specifically examined the HG duplication patterns in other neuropsychiatric disorders, such as affective disorders, that partly overlap with schizophrenia on the level of phenomenology and genetic/neurobiological findings (Prata et al., 2019; Grunze and Cetkovich-Bakmas, 2021).

While the neural underpinnings of affective disorders remain elusive, it is hypothesized that affective disorders, particularly bipolar disorder (BD), may be caused by developmentally mediated neurobiological alterations that are associated with emotion-regulation neural circuitry (Sanches et al., 2008; Phillips and Swartz, 2014). Major depressive disorder (MDD) is a phenotypically heterogeneous disorder with both biological and environmental risk factors (Slavich and Irwin, 2014; Uchida et al., 2018), in addition to which prenatal neurodevelopmental insults may also contribute to its pathophysiology (Gałecki and Talarowska, 2018; Lima-Ojeda et al., 2018). Indeed, previous MRI studies in schizophrenia (Takahashi et al., 2014b; Nishikawa et al., 2016), BD (Takahashi et al., 2014a), and MDD (Takahashi et al., 2016) have demonstrated commonly altered brain surface morphology, suggesting partly overlapping neurodevelopmental pathologies in these disorders. Further, it is notable that schizophrenia and BD patients likely exhibit similar gyrification pattern trajectories (reviewed by Sasabayashi et al., 2021) as a potential common basis of emotional dysregulation and cognitive impairments. Given that inter-individual variation in the HG gyrification pattern could affect regional neural functions and cognitive abilities (Tzourio-Mazoyer et al., 2015; Tzourio-Mazoyer and Mazoyer, 2017) and that the HG is also involved in emotional processing (Grosso et al., 2015; Concina et al., 2019), it would seem worthwhile to evaluate the potential role of HG duplication patterns on the pathophysiology of affective disorders.

The present MRI study aimed to examine the HG duplication patterns in both BD and MDD in comparison with our previous findings in schizophrenia (Takahashi et al., 2021a,b,c) to establish the common and distinct alterations in HG gyrification pattern across major psychiatric disorders. On the basis of the potential role of HG patterns in emotional processing (e.g., Tzourio-Mazoyer and Mazoyer, 2017) and previous findings of partly overlapping brain gyrification patterns in various psychiatric disorders (Sasabayashi et al., 2021), we predicted that affective disorders (especially BD) would have an increased HG duplication compared to matched healthy controls. We also explored the relationship between HG patterns and clinical characteristics in the BD and MDD groups.

## Materials and Methods

### Participants

The study participants comprised 26 patients with BD, 56 with MDD, and 57 age- and sex-matched healthy controls (24 subjects matched for BD and 33 for MDD) (Table 1); inclusion/exclusion criteria and sample characteristics of these cohorts have been fully described elsewhere (Takahashi et al., 2014a,^2016^, ^2020^).

**TABLE 1:** Sample characteristics of the study participants.

	BD cohort		MDD cohort	
	Controls (*N* = 24)	Patients (*N* = 26)	Controls (*N* = 33)	Patients (*N* = 56)
Age (years)	38.7 ± 11.1	38.4 ± 10.9	34.0 ± 9.9	33.8 ± 9.1
Male/female	7/17	8/18	12/21	16/40
Current IQ	115.1 ± 9.6	113.8 ± 7.1	111.1 ± 10.9	108.0 ± 9.8
Age of onset (years)	−	24.9 ± 8.4	−	23.5 ± 9.0
Illness duration (years)	−	13.5 ± 10.1	−	10.3 ± 8.1
Number of manic episodes	−	8.8 ± 10.2	−	−
Number of depressive episodes	−	11.1 ± 10.8	−	3.4 ± 3.0
Medication at scanning (yes/no)	−	21/5	−	33/19
Beck Depression Inventory	−	−	3.6 ± 4.1	23.4 ± 15.8
MASQ general distress	−	−	27.9 ± 8.3	45.8 ± 10.3
MASQ general depression	−	−	19.5 ± 7.2	41.5 ± 12.0
MASQ general anxiety	−	−	16.4 ± 6.4	28.7 ± 9.0
MASQ anxious arousal	−	−	22.0 ± 4.4	36.1 ± 12.2
MASQ high positive affect	−	−	81.1 ± 14.3	53.5 ± 16.8
MASQ loss of interest	−	−	14.7 ± 5.0	27.8 ± 7.7
PANAS positive affect	−	−	32.9 ± 7.3	25.0 ± 8.0
PANAS negative affect	−	−	11.2 ± 1.6	17.8 ± 7.7

*Values represent means ± SD unless otherwise stated. BD, bipolar disorder; MASQ, Mood and Anxiety Symptom Questionnaire; MDD, major depressive disorder; PANAS, Positive and Negative Affect Schedule.*

Briefly, the patients fulfilling DSM-IV criteria for bipolar I disorder were recruited from the Mood Disorders Unit at the Prince of Wales Hospital, Sydney, Australia. Their diagnoses and clinical characteristics (e.g., lifetime affective episodes, medication status) were confirmed by research psychiatrists using the Structured Clinical Interview for DSM-IV patient version (SCID-IV-P) (First et al., 1998) and a detailed case note review. At the time of participation, all patients did not fulfill current manic/hypomanic or depressive episode of SCID and were considered to be under euthymic condition only with subsyndromal symptoms. Twenty-one patients were taking mood stabilizers [e.g., lithium (Li) (*N* = 12), valproate (VPA) (*N* = 12)], while the remaining 5 were not on medication at the time of scanning. Ten BD patients had a family history of affective disorders and 16 had a history of psychosis (i.e., hallucinations and/or delusions) during past affective episodes.

The MDD patients were recruited *via* local advertisement or outpatient psychiatric clinics in Melbourne, Australia. They were diagnosed by SCID-IV-P (First et al., 1998) and assessed using the Beck Depression Inventory (BDI) (Beck and Steer, 1987), Positive Affect and Negative Affect Scale (PANAS) (Watson et al., 1988), and Mood and Anxiety Symptom Questionnaire (MASQ) (Watson et al., 1995) by experienced research psychologists at ORYGEN Youth Health, Melbourne. At that time, medication status in the preceding 6 months of the study was also assessed through direct interview and medical record review. At the time of scanning, 29 patients fulfilled DSM criteria of MDD (i.e., currently depressed), while 27 had a history of MDD but currently in remission. Twenty-two MDD patients (18 currently depressed and 4 remitted patients) had a comorbid diagnosis of anxiety disorders.

Participants were right-handed and were screened for head trauma, neurological illness, substance misuse, or other serious physical diseases. Age- and sex-matched healthy comparison subjects for BD (Sydney) and MDD (Melbourne) groups, screened for a personal or family history of psychiatric diseases using the SCID-IV non-patient version (First et al., 1998), were recruited through local advertisement. The study protocol was approved by the local Internal Review Boards (the Prince of Wales Hospital and University of New South Wales research ethics committees and Mental Health Research and Ethics Committee, Melbourne Health, Melbourne, Australia). The participants provided written informed consent after a complete description of the study in accordance with the Declaration of Helsinki.

### Magnetic Resonance Imaging Procedures

Bipolar disorder patients and their comparison subjects were scanned using a 1.5-T GE Signa scanner at Royal Prince Alfred Hospital, Sydney, Australia, where a fast-spoiled gradient echo sequence was applied to obtain T1-weighted consecutive coronal images with a voxel size of 0.98 mm × 0.98 mm × 1.6 mm. MDD patients and their controls were scanned by a1.5T Siemens scanner (Magnetom Avanto) at Saint Vincent’s Hospital Melbourne, Victoria and T1-weighted iso-voxel (1.0 mm × 1.0 mm × 1.0 mm) images were obtained in the axial orientation. Detailed imaging parameters for the BD and MDD cohorts are available elsewhere (Takahashi et al., 2014a,^2016^, ^2020^).

**FIGURE 1:**
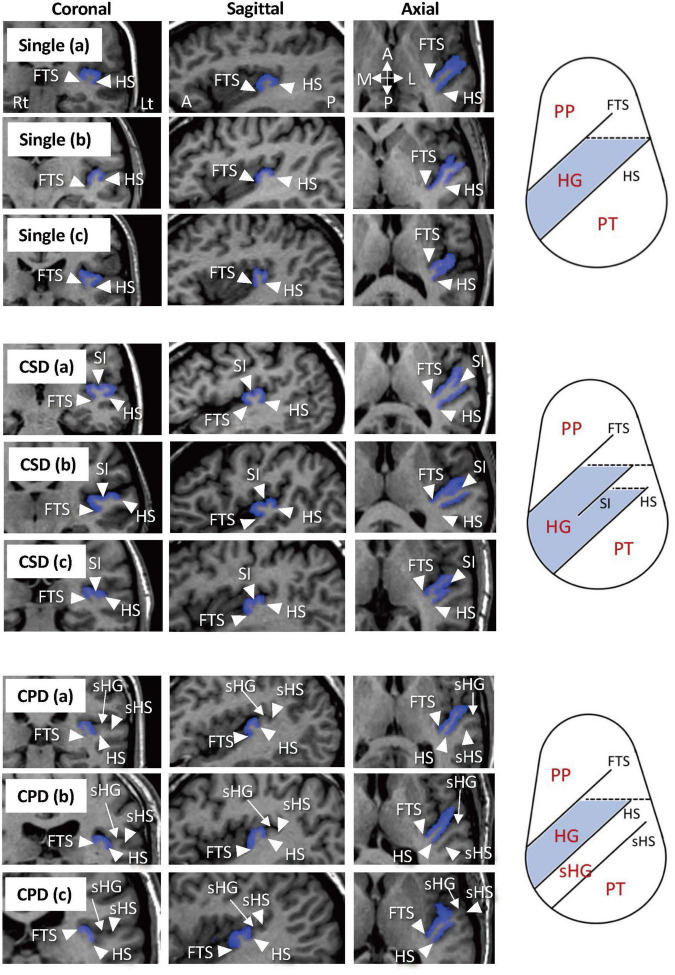
Sample images of various Heschl’s gyrus (HG) patterns and anatomical landmarks on MR images and on pattern diagrams in axial direction. The HGs on the left hemisphere are colored in blue. Subjects with a single HG pattern sometimes had a small branching at the front tip [Single (b)] (Marie et al., 2015) or a shallow cortical dimple at the crown of the HG [Single (c)]. Two hemispheres in the present study that had a separate HG posterior to the HG with partial duplication were considered to have the CSD pattern [CSD (c)]. One subject had a pattern of three separate HGs in the left hemisphere, which was classified as a variation of CPD [CPD (c)]. A, anterior; CPD, complete posterior duplication; CSD, common stem duplication; FTS, first transverse sulcus; HS, Heschl’s sulcus; L, lateral; P, posterior; M, medial; PP, planum polare; PT, planum temporale; sHG, second Heschl’s gyrus; sHS, second Heschl’s sulcus; SI, sulcus intermedius.

For the assessment of HG gyrification patterns, brain images were realigned in three dimensions, followed by reconstruction into entire 0.98-mm (BD cohort)- or 1-mm (MDD cohort)-thick contiguous coronal images that were perpendicular to the anterior commissure-posterior commissure line using Dr. View software (Infocom, Tokyo, Japan). As fully described previously (Takahashi et al., 2021a,b,c), one experienced rater with no knowledge of the subjects’ identity (TT) classified the HG gyrification into single, partly duplicated (i.e., common stem duplication; CSD), or completely duplicated (i.e., complete posterior duplication; CPD) pattern. While brain images were not corrected for inhomogeneity/artifact, anatomical landmarks for the classification were readily identified by referring to images from three directions all together (Figure 1). Another rater (DS), who was also experienced for HG pattern classification (Takahashi et al., 2021a,b,c), independently classified the HG patterns in a subset of randomly selected 15 brains (30 hemispheres). Intra- (TT) and inter-rater (TT and second-rater DS) reliabilities were 30/30 agreement (Cronbach’s α = 1.00) and 29/30 agreement (Cronbach’s α = 0.87), respectively.

### Statistical Analysis

Group differences in the HG pattern distribution were tested by the χ^2^ test or Fisher’s exact test when more than 20% of cells had expected counts <5.

Non-parametric Mann-Whitney U tests were used for assessing the relationship between the HG patterns and clinical variables, because of the non-normal distribution of most of these variables and small sample size for each HG pattern. The CSD and CPD patterns were categorized together as the ‘duplicated pattern’ here also due to small sample size for each pattern. Potential role of HG patterns on symptom ratings in MDD was assessed separately on the currently depressed and remitted subgroups because these subgroups were highly different in symptom severity. Statistical significance was set at *p*-value < 0.05.

## Results

### Sample Characteristics

The BD and MDD groups did not differ to their controls in terms of age, sex, and intelligence (Table 1). Currently depressed and remitted MDD subgroups did not differ for these demographic variables, while the currently depressed group had more severe depressive/anxiety symptoms and higher medication rates than the remitted group (Takahashi et al., 2016, 2020).

### Heschl’s Gyrus Pattern Distribution

**FIGURE 2:**
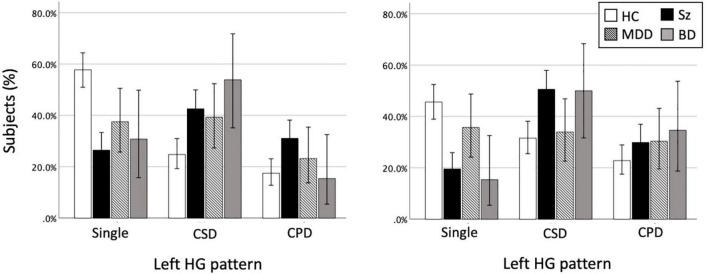
Heschl’s gyrus (HG) gyrification patterns in healthy controls (HC), schizophrenia (Sz), major depressive disorder (MDD), and bipolar disorder (BD). The present study examined the HG patterns in MDD and BD cohorts, but the data of 174 patients with Sz (Takahashi et al., 2021a,b,c) are also presented here for the purpose of comparison. The results of the HC group (*N* = 206) reflect all data from the present and our previous (Takahashi et al., 2021a,b,c) studies. Direct comparisons between the disorders showed that the MDD patients had a lower prevalence of right HG duplication compared to Sz (χ^2^ = 6.17, *p* = 0.013) and BD (χ^2^ = 3.55, *p* = 0.060) patients. However, there was no group difference between the BD and Sz. Error bars show 95% confidence intervals. CPD, complete posterior duplication; CSD, common stem duplication.

The BD patients had a higher prevalence of HG duplication for both left (χ^2^ = 6.44, *p* = 0.011) and right (χ^2^ = 5.51, *p* = 0.019) hemispheres compared to controls, but there was no group difference when only the participants with duplicated HG were examined (i.e., CSD *vs.* CPD) (Table 2 and Figure 2).

**TABLE 2:** Gyrification pattern of Heschl’s gyrus (HG) for both hemispheres in the bipolar disorder (BD) cohort.

		Right HG pattern [*N* (%)]			
		Single	CSD	CPD	Total
Healthy controls					
Left HG pattern [*N* (%)]	Single	8 (33.3)	4 (16.7)	4 (16.7)	16 (66.7)
	CSD	0 (0)	3 (12.5)	1 (4.2)	4 (16.7)
	CPD	3 (12.5)	1 (4.2)	0 (0.0)	4 (16.7)
	Total	11 (45.8)	8 (33.3)	5 (20.8)	24 (100.0)
**BD**					
Left HG pattern [*N* (%)]	Single	2 (7.7)	2 (7.7)	4 (15.4)	8 (30.8)
	CSD	2 (7.7)	9 (34.6)	3 (11.5)	14 (53.8)
	CPD	0 (0)	2 (7.7)	2 (7.7)	4 (15.4)
	Total	4 (15.4)	13 (50.0)	9 (34.6)	26 (100.0)

*CSD, common stem duplication; CPD, complete posterior duplication.*

No significant group difference was observed between the MDD patients and matched controls irrespective of HG classification (i.e., whether CSD and CPD patterns were grouped or not) (all *p* > 0.117; Table 3 and Figure 2).

**TABLE 3:** Gyrification pattern of Heschl’s gyrus (HG) for both hemispheres in the major depressive disorder (MDD) cohort.

		Right HG pattern [*N* (%)]			
		Single	CSD	CPD	Total
Healthy controls					
Left HG pattern [*N* (%)]	Single	9 (27.3)	6 (18.2)	3 (9.1)	18 (54.5)
	CSD	4 (12.1)	1 (3.0)	2 (6.1)	7 (21.2)
	CPD	4 (12.1)	1 (3.0)	3 (9.1)	8 (24.2)
	Total	17 (51.5)	8 (24.2)	8 (24.2)	33 (100.0)
**cMDD**					
Left HG pattern [*N* (%)]	Single	5 (17.2)	1 (3.4)	4 (13.8)	10 (34.5)
	CSD	5 (17.2)	4 (13.8)	1 (3.4)	10 (34.5)
	CPD	1 (3.4)	4 (13.8)	4 (13.8)	9 (31.0)
	Total	11 (37.9)	9 (31.0)	9 (31.0)	29 (100.0)
**rMDD**					
Left HG pattern [*N* (%)]	Single	3 (11.1)	4 (14.8)	4 (14.8)	11 (40.7)
	CSD	3 (11.1)	6 (22.2)	3 (11.1)	12 (44.4)
	CPD	3 (11.1)	0 (0.0)	1 (3.7)	4 (14.8)
	Total	9 (33.3)	10 (37.0)	8 (29.6)	27 (100.0)

*cMDD, currently depressed patients; CSD, common stem duplication; CPD, complete posterior duplication; rMDD, remitted depressed patients.*

The two independent control groups (24 subjects for BD and 33 for MDD) did not differ in HG pattern distribution. While sex may affect cortical folding developments (Mutlu et al., 2013), we found no significant sex difference in the HG patterns.

### Relationship Between the Heschl’s Gyrus Pattern and Clinical Characteristics

For both the BD and MDD patients, the HG patterns did not relate to age of onset, illness duration, number of affective episodes, or medication status (yes/no for MDD, Li-treated *vs.* non-Li-treated and VPA-treated *vs.* non-VPA-treated for BD). Also, psychotic symptoms and family history of affective disorders in the BD patients were not associated with the HG patterns.

For the currently depressed MDD patients, the patients with single HG had more severe depressive/anxiety symptoms than those with HG duplication especially for the right hemisphere (Table 4). However, remitted MDD patients showed no relationship between the HG patterns and these symptom ratings. For the MDD group as a whole, the patients with right single HG had a higher rate of comorbid anxiety disorder than those with right duplicated HG (χ^2^ = 5.24, *p* = 0.022).

**TABLE 4:** Symptom ratings of the currently depressed patients with different Heschl’s gyrus (HG) patterns.

	Left hemisphere			Right hemisphere		
	Single HG (*N* = 10)	Duplicated HG (*N* = 19)	Mann-Whitney tests	Single HG (*N* = 11)	Duplicated HG (*N* = 18)	Mann-Whitney tests
Beck Depression Inventory	41.5 ± 8.2	34.4 ± 8.5	*U* = 53.0, *p* = 0.056	41.6 ± 6.2	33.9 ± 9.2	*U* = 44.0, *p* = 0.012
MASQ general distress	50.9 ± 6.2	50.3 ± 8.7	*U* = 87.5, *p* = 0.906	55.7 ± 5.4	47.1 ± 7.3	*U* = 31.5, *p* = 0.002*^a^*
MASQ general depression	53.3 ± 5.7	44.0 ± 9.2	*U* = 37.5, *p* = 0.010	51.6 ± 6.8	44.5 ± 9.6	*U* = 46.0, *p* = 0.025
MASQ general anxiety	31.8 ± 6.9	32.4 ± 9.8	*U* = 95.0, *p* = 0.832	40.0 ± 5.2	27.1 ± 6.5	*U* = 10.5, *p* < 0.001*^a^*
MASQ anxious arousal	41.9 ± 7.8	42.1 ± 14.2	*U* = 92.0, *p* = 0.944	49.7 ± 11.5	37.0 ± 10.0	*U* = 38.0, *p* = 0.008
MASQ high positive affect	34.5 ± 5.8	48.6 ± 14.0	*U* = 146.0, *p* = 0.006	41.6 ± 10.1	44.9 ± 15.4	*U* = 101.5, *p* = 0.711
MASQ loss of interest	34.7 ± 7.3	29.9 ± 5.2	*U* = 39.5, *p* = 0.014	35.0 ± 5.2	29.4 ± 6.2	*U* = 46.0, *p* = 0.025
PANAS positive affect	19.8 ± 5.6	22.5 ± 6.8	*U* = 107.0, *p* = 0.308	20.3 ± 6.6	22.5 ± 6.4	*U* = 117.5, *p* = 0.264
PANAS negative affect	23.1 ± 9.6	20.3 ± 8.0	*U* = 71.0, *p* = 0.498	28.4 ± 7.8	16.6 ± 5.1	*U* = 22.0, *p* < 0.001*^a^*

*Values represent means ± SD. MASQ, Mood and Anxiety Symptom Questionnaire; PANAS, Positive and Negative Affect Schedule.*

*^a^Significant even after Bonferroni’s correction for multiple comparisons [18 comparisons; p < 0.00278 (0.05/18)].*

For the Melbourne healthy controls, who were assessed for depressive and anxiety ratings, the subjects with right HG duplication had a higher MASQ anxious arousal score (mean = 24.1, SD = 5.5) than those with right single HG (mean = 20.0, SD = 1.5) (*U* = 185.5, *p* = 0.008).

## Discussion

This MRI study in affective disorders (BD and MDD) examined the disease specificity of the HG gyrification patterns in comparison with previous findings in schizophrenia, because these major neuropsychiatric disorders exhibit partly common phenomenology (e.g., depressive symptoms in BD and MDD, executive dysfunction in BD and schizophrenia) and brain characteristics associated with gyrification pattern (reviewed by Sasabayashi et al., 2021). One of the strengths of this study is that it includes both MDD and BD cohorts, as differences/similarities of brain morphology between these affective disorders have not been well explored. Our results demonstrated that the BD patients had an increased prevalence of HG duplication bilaterally, which was similar to our previous findings in schizophrenia (Takahashi et al., 2021a,b,c). While the main objective of this study was to show the prevalence of HG duplication in affective disorders, we also explored potential contribution of HG patterns on clinical characteristics. The MDD patients did not differ in the prevalence of HG duplication compared to healthy controls, but their HG patterns were significantly associated with symptom severity during a depressive episode. These findings suggest partly overlapping neurodevelopmental origins between BD and schizophrenia, while the neurodevelopmental process associated with embryonic gyral formation may also contribute to certain clinical aspects of MDD. While we have previously reported a reduced normal leftward volumetric asymmetry of the planum temporale, which locates directly posterior to HG, in both BD (Takahashi et al., 2010a) and MDD (Takahashi et al., 2010b) groups as a common gross morphologic feature, the present results suggest the specific role of HG patterns as a distinct marker between these affective disorders.

The present finding of increased prevalence of duplicated HG in the BD patients is in line with the notion of common neurobiological substrates for BD and schizophrenia (Goodkind et al., 2015), a hypothesis that has been supported by a wide range of similarities in genetic (Lichtenstein et al., 2009; Bipolar Disorder and Schizophrenia Working Group of the Psychiatric Genomics Consortium, 2018; Brainstorm et al., 2018), neuroimaging (Hanford et al., 2016; Koshiyama et al., 2020), and neuropsychological (Bora, 2015) findings. The inter-individual variations in the HG gyrification are formed during late gestation along with neural development (Chi et al., 1977; Van Essen, 1997) and its duplication may lead to learning disability after birth (Leonard et al., 1993, 2001), and regional dysfunction in adulthood (Tzourio-Mazoyer et al., 2015). The HG is a part of the primary auditory cortex (Rademacher et al., 1993; Da Costa et al., 2011) but it also plays a crucial role in emotional processing (Grosso et al., 2015; Concina et al., 2019). Interestingly, recent neuroimaging studies have demonstrated shared glutamatergic abnormalities (Atagün et al., 2015), reduced cortical thickness (Mørch-Johnsen et al., 2018), and reduced functional connectivity (Wei et al., 2018) in BD and schizophrenia patients in the HG region. Taken together with these findings, our results likely support the hypothesis that BD and schizophrenia patients exhibit shared hyper-gyrification and compromised neural connectivity in the cortical regions as a consequence of pre/perinatal neurodevelopmental insult, which later underpin common clinical manifestations such as emotional dysregulation and executive dysfunction (Sasabayashi et al., 2021). Our results further revealed no relationship between the HG patterns and illness stages and medication status in the BD patients, supporting its role as a stable trait marker.

In contrast to the findings in BD and schizophrenia (Takahashi et al., 2021a,b,c), the HG patterns in the MDD patients did not differ significantly from those of healthy controls, suggesting a less prominent neurodevelopmental pathology. Previous transdiagnostic studies in brain gyrification of temporal region (Sasabayashi et al., 2021) and white matter microstructure in the limbic system (Koshiyama et al., 2020) also demonstrated near-normal findings only in the MDD among these disorders. On the other hand, we found a significant relationship between the single HG pattern and severe depressive/anxiety symptoms in the MDD patients under an active depressive state. This relationship was somewhat unexpected because the HG duplication, which may relate to regional dysfunction (Tzourio-Mazoyer et al., 2015), contributed to anxiety tendencies in healthy subjects in this study. However, a recent MRI study in MDD also suggested potential contribution of hypo-gyrification to depressive symptomatology in various regions of the brain (Schmitgen et al., 2019). Since this structural MRI study cannot address the functional significance of the HG patterns on depression symptomatology, potential mechanisms of different contribution of HG patterns on anxiety between non-clinical population and pathological status remains unknown and should be examined in future studies exploring this relationship. Normal or even higher prevalence of single HG in the tinnitus patients compared to controls (Schneider et al., 2009) may also support a complex relationship between the HG patterns and regional functioning. Nevertheless, the present study suggested that embryonic neurodevelopmental processes associated with gyral formation of HG may play a role in the phenomenology of MDD in later life potentially by interacting with environmental factors in the epigenetic mechanisms (Gałecki and Talarowska, 2018).

It should be noted that HG duplication itself is observed in healthy subjects and is associated with their cognitive abilities (Marie et al., 2016). In particular, musical ability in subjects without neuropsychiatric disorders seems to be associated with larger HG (Schneider et al., 2002; Seither-Preisler et al., 2014; Wengenroth et al., 2014; Dalboni da Rocha et al., 2020) and higher percentage of HG duplications (Schneider et al., 2005; Benner et al., 2017) especially on the right hemisphere. Because individuals with William Beuron syndrome, a rare genetic disorder with characteristic musicality, likely exhibit larger HG and increased HG duplication predominantly on the left hemisphere (Wengenroth et al., 2010), it may be hypothesized that changes in the right and left HGs associated with musicality may be mainly attributable to the amount of training and genetic factors, respectively. It is currently unknown whether increased HG duplication in the neuropsychiatric disorders has different mechanisms from inter-individual HG variation in healthy subjects, but the former probably reflects their early neurodevelopmental pathology. Given that right HG generally develops 1 to 2 weeks earlier than left HG during mid-to-late gestation (Chi et al., 1977), our results of bilateral changes in HG pattern in schizophrenia (Takahashi et al., 2021a,b,c) and BD may support severe and prolonged neurodevelopmental abnormalities in these disorders. Further, schizophrenia (Takahashi et al., 2021a) and BD (Takahashi et al., 2010a) groups have an increased HG duplication with marked HG ‘atrophy,’ suggesting different mechanisms between normal variation in the HG morphology and HG changes in these neuropsychiatric disorders.

Several potential confounding factors in this study should be noted. First, different MR settings (e.g., scanners, parameters) used for the BD and MDD patients limited the comparability of our data (Pøibil et al., 2019). We therefore used the control groups matched for demographic background and MR setting for each patient group. Further, it is unlikely that different scanning condition significantly affected our conclusion, because the anatomical landmarks for HG classification (Figure 1) could be readily identified in all of the study participants. In this study, we referred to our previous results in schizophrenia (Takahashi et al., 2021a,b,c) to interpretate the current findings in affective disorders. However, these previous data were assessed in different racial/ethnic population (Toyama, Japan) from the current Australian cohorts, which might affect the results (Brickman et al., 2008; Rao et al., 2017). Although we found no significant differences in HG pattern distribution at least between three control groups with different MR settings and populations (Sydney, Melbourne, and Toyama), future transdiagnostic studies with more homogeneous conditions (i.e., on a single MRI scanner) are required. Second, the sample size of both disease groups and healthy controls was relatively small, which may have contributed to the lower statistical power. While the MDD patients showed no significant difference in HG patterns compared to controls, they were characterized by a somewhat higher duplication rate especially on the left hemisphere (Figure 2). Because the HG may also participate in learning and memory processing (Weinberger, 2015), it may be possible that future study in a larger MDD cohort will detect an altered HG pattern as a common neural underpinning of memory deficits observed in MDD, BD, and schizophrenia (Marazziti et al., 2010; Esan et al., 2020). Finally, it was not possible to examine the relationship between the HG patterns and symptom severity in our BD cohort because they were under remission state at the time of scanning. Further, despite potential contribution of HG gyrification patterns to cognitive function for both non-clinical population (Tzourio-Mazoyer et al., 2015) and schizophrenia (Takahashi et al., 2021c), the current BD and MDD patients were not systematically assessed for their cognitive impairment. Thus, the potential role of HG patterns on the phenomenology of affective disorders (especially symptom severity and cognitive function in BD) and its disease specificity requires further exploration.

In conclusion, the present study demonstrated that patients with BD have a common macroscopic brain characteristic of increased HG duplication with those who have schizophrenia, which may partly underlie common clinical manifestations between these disorders. Conversely, the distribution of HG patterns in the MDD patients was similar to healthy controls and distinctively different from these disorders. While replication studies in a larger transdiagnostic cohort will be clearly required, our results of distinct HG patterns between the BD and MDD patients may contribute to imaging-based differential diagnosis and prediction of clinical course (e.g., later manic episode) at early stages in patients with depressive symptoms.

## Data Availability Statement

The raw data supporting the conclusion of this article will be made available by the authors, without undue reservation.

## Ethics Statement

The studies involving human participants were reviewed and approved by the Prince of Wales Hospital and University of New South Wales Research Ethics Committees and Mental Health Research and Ethics Committee, Melbourne Health, Melbourne, Australia. The patients/participants provided their written informed consent to participate in this study.

## Author Contributions

MY, MS, CP, GM, and NA conceived the concept for and methodology of the study and contributed to the writing and editing of the manuscript. TT conducted statistical analyses and wrote the manuscript. MY, SW, VL, MW, GM, and NA recruited subjects and were involved in clinical and diagnostic assessments. TT and DS analyzed MRI data. All authors contributed to and have approved the final manuscript.

## Conflict of Interest

The authors declare that the research was conducted in the absence of any commercial or financial relationships that could be construed as a potential conflict of interest.

## Publisher’s Note

All claims expressed in this article are solely those of the authors and do not necessarily represent those of their affiliated organizations, or those of the publisher, the editors and the reviewers. Any product that may be evaluated in this article, or claim that may be made by its manufacturer, is not guaranteed or endorsed by the publisher.
